# 1813. A Population-Based Multi-Year Assessment of the Incidence of High Inoculum Cefazolin (CZ) Resistance Amongst Methicillin-Susceptible *Staphylococcus aureus* (MSSA) Causing Bloodstream Infections (BSIs) over time in Calgary, Canada

**DOI:** 10.1093/ofid/ofac492.1443

**Published:** 2022-12-15

**Authors:** Kristine Du, Barbara Jean M Waddell, Chloe Ligier, Julianna Svishchuk, Alexander Kipp, John Lam, Stephen Robinson, Ranjani Somayaji, John M Conly, Daniel Gregson, Michael Parkins

**Affiliations:** University of Calgary, Calgary, Alberta, Canada; University of Calgary, Calgary, Alberta, Canada; University of Calgary, Calgary, Alberta, Canada; University of Calgary, Calgary, Alberta, Canada; University of Calgary, Calgary, Alberta, Canada; University of Calgary, Calgary, Alberta, Canada; Dalhousie University, Saint John, New Brunswick, Canada; University of Calgary, Calgary, Alberta, Canada; University of Calgary, Calgary, Alberta, Canada; University of Calgary, Calgary, Alberta, Canada; University of Calgary, Calgary, Alberta, Canada

## Abstract

**Background:**

Cefazolin (CZ) is a 1^st^-line therapy for methicillin-susceptible *Staphylococcus aureus* (MSSA) infections due to its tolerability, lower toxicity, and convenient dosing. However, some strains exhibit reduced susceptibility to CZ when present in high inoculum. Treatment failure and worsened outcomes are documented in BSI cohorts treated with CZ when infections are caused by strains demonstrating this inoculum effect (IE) (Miller, 2018). The objective of our study was to investigate the incidence of MSSA strains demonstrating IE over time across a general population, free of referral bias.

**Methods:**

All initial isolates of first episodes of MSSA BSI from persons in Calgary, Canada were analyzed during 2012-14 and 2019. CZ minimum inhibitory concentration (MIC) was determined at standard (SI: 5x10^5^ CFU/mL) and high inocula (HI: 5x10^7^ CFU/mL) using the broth microdilution method at 24 hours as per 2009 CLSI criteria. Isolates were defined as having the CZ IE if they had a ≥ 4-fold difference between MIC at SI and HI. Isolates were defined as having a *pronounced IE* (pIE), if they were susceptible at SI but resistant at HI with an MIC ≥ 32µg/mL. Population density of Calgary was obtained from census data. Annual incidence rates (IR) were calculated by dividing the number of IE and pIE by the mid-year population data, and IR ratios (IRR) were calculated for IE and pIE by comparing 2019 to 2012 rates.

**Results:**

During the years of study, 1040 individuals experienced at least one episode of MSSA BSI. The mean incidence rates of IE and pIE were 6.50 and 0.98 per 100,000 person-years, respectively. Comparing 2019 to 2012, rates of IE decreased significantly with an IRR of 0.67 (95% CI 0.47 – 0.95) but rates of pIE were not significantly different (IRR 1.24; 95% CI 0.43 – 3.86).

Incidence rates of IE and pIE in Calgary, Canada in 2012-14 and 2019.

**Figure:**
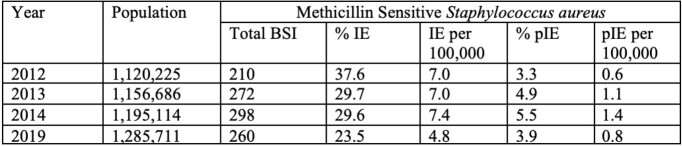

MSSA BSIs in Calgary, Canada over the years are shown alongside corresponding rates of IE and pIE. The population density was used to find rates for IE/pIE per 100,000 person-years.

**Conclusion:**

Population-based studies on MSSA BSI with the IE and pIE phenotype are lacking. Herein, we observed that while the incidence of MSSA with pIE has not changed, those occurring with the IE have decreased. Further studies on the clinical implications of pIE are needed.

**Disclosures:**

**All Authors**: No reported disclosures.

